# Notes and News

**Published:** 1887-04-30

**Authors:** 


### Notes and News.

Collected and Communicated.

Mr. John Wood has been elected to the post of consulting surgeon to the Brighton Dental Hospital.

Mr. Henry White, late of the Warehousemen and Clerks' Schools, has been appointed Secretary to the Metropolitan Free Hospital, Kingsland Road.

The post of matron at the Hospital for Epilepsy and Paralysis, and other Diseases of the Nervous System, Regent's Park, has been filled by the appointment of Miss Foster, Superintendent of nurses at St. Pancras Infirmary, and formerly Matron of the Maidenhead Cottage Hospital.

Out of over thirty sets of designs for the new hospital at Derby, submitted in public competition, that of Messrs. Ellison and Son, of Liverpool, has been selected by the assessor. The design of Mr. M. R. Evans, of the Corn Market, Derby, has been placed second, entitling the architect to a premium of £20.

The *City Press* remarks :—" Some little doubt seems to exist in the minds of the public as to the identity of Mr. Morgan, who has promised £10,000, conditionally, to Guy's Hospital. He is the nominal successor in Mr. Peabody's business in Leadenhall Street, and, as the Lord Mayor truly says, is his worthy successor in philanthropic work."

The unfavourable weather which has of late years proved so serious an obstacle to the success of Hospital Sunday in Liverpool has caused the promoters to seriously consider the advisability of changing the date on which these collections are made. Hitherto Hospital Sunday in Liverpool has always been held in January, and this year many people were kept indoors by a heavy fall of snow. Endeavours are about to be made to obtain the opinions of the clergy and ministers of the various churches and chapels in which collections have been held as to the most suitable date to select. On this point there is some dispute, as the advocates of the existing system point out that in the summer many families are away, and there would then be, in their view, a greater danger of reduction in the sums collected than at present.

A new hospital for infectious diseases has recently been opened at Burslem. For some years past, the need of a sanatorium has been much felt, but various causes have hitherto prevented its erection. The total cost of the new hospital, including the site, has been £4,204 19*s.* 5*d.* It has been designed by Mr. G. B. Ford, architect, of Burslem, and erected by Mr. Webb, of Silverdale. The sanatorium is a handsome structure, built in five separate blocks.

The first annual meeting of the friends of the Liverpool Foundling Hospital was held on the 4th April, at the Town Hall, under the presidency of the Mayor. The institution has been at work in Upper Parliament Street for about nine months. During that period eight infants have been admitted, and two (whose mothers have since returned to domestic service) have been born in the hospital. There have been two deaths. Eight infants are now in the hospital, and several applications for admission are under consideration. There is accommodation for about twenty children and for three or four confinement cases at one time ; but it is thought that, if need arises, there will be no great difficulty in enlarging the premises. The Committee intend to place out the children at the earliest possible age (in some cases at four or five years old), by emigration and other means, so that as far as possible the hospital may be constantly available for new and deserving cases.

The Jubilee Extension Fund of the Aberdeen Royal Infirmary now amounts to about £19,000.——A Jubilee Cot Hospital, to contain five beds, is to be built at Wimborne, at a cost of about £950. The honorary secretary, the Rev. E. E. Cleal, has collected about £1,060 for this purpose.——A site has been selected for the Hornchurch Jubilee Cottage Hospital, and a sum of £411 has been collected by the honorary secretary, while £94 3*s.* has been promised as annual subscriptions.——In commemoration of the fiftieth year of Her Majesty's reign, a Convalescent Home is to be erected in connection with the Belfast Hospital for Sick Children. The town is being actively canvassed for subscriptions.——The Duke of Northumberland has generously offered to give a site for the new Tynemouth Infirmary. The building fund now amounts to £800, and plans of the new building have been prepared.——The Princess of Wales and the Duchess of Teck have kindly consented to become patronesses of the Children's Jubilee Tribute movement, the object of which is to raise funds for the erection and endowment of a new wing to the Great Ormond Street Children's Hospital, the oldest Children's Hospital in the Metropolis.——A "Queen Victoria Jubilee Endowment Fund" has been started to provide an increased income for the Cumberland Infirmary.——The County and City of Dublin Committee for the celebration of the Queen's Jubilee have adopted a report from the Executive Committee, recommending the erection in the county of a Hospital for Consumption, at an estimated cost of £12,000.——A Jubilee Cottage Hospital is projected for Mexborough, to contain from six to twelve beds.——In commemoration of the Queen's Jubilee, the residents of Eastbourne have determined to enlarge the Princess Alice Memorial Hospital.

The following vacancies are announced this week, the amount of the salary being placed in each case after the name of the institution :—

**Honorary Medical Officer.**

Farringdon Dispensary. Physician. University Graduate.

**Resident Medical Officers.**

Brighton Alexandra Hospital for Children. House Surgeon. Doubly-qualified.—£80.
Central London Ophthalmic Hospital. House Surgeon and Assistant Surgeon. Qualified.
Cheltenham Hospital. Assistant Surgeon. Doubly-qualified. —£40.
Dorset County Asylum. Assistant. Doubly-qualified.—£120.
Great Northern Central Hospital. House Physician. Qualified.
Horton Infirmary. House Surgeon. Doubly-qualified.—£60.
Metropolitan Free Hospital. Assistant House Surgeon.
Stafford County Asylum. Superintendent.—£700.
Stanhope Street Dispensary, Clare Market. One. Doubly-qualified.—£105.

**Non-resident Medical Officers.**

Birmingham, Mason College. Professor of Physiology.
Dental Hospital of London. Assistant Surgeon. Licentiate.

**Lady Superintendent.**

Newcastle Eye Infirmary.

**Trained Nurses.**

Croydon Nurses' Institute. Several.—£20.
Cumberland and Westmoreland Convalescent Institution, Silloth. One.—£30.
Dorset County Hospital. Head.—£25. Assistant.—£16.
Mavisbank Asylum, near Edinburgh. Head Nurse and Companion.—£25.
Mold Cottage Hospital. One.—£25.
Newcastle, St. Mary Magdalene Home. One.—£20.
New Hospital for Women, 222, Marylebone Road, N.W.—Commencing at £18.
Yeovil Hospital. One.—£30.

**Probationer.**

Ancoats Hospital, Manchester. One.

---

On Saturday evening a meeting was held at the Morley Hall, Bow, by the representatives of the various Trade, Friendly, Temperance, and Benefit Societies of the East End of London in support of a scheme for the liquidation of the debt now existing upon the Morley House Seaside Convalescent Home, at St. Margaret's, near Dover. Mr. J. T. Medhurst presided. Mr. Robert Frewer, the Secretary of the Hospital Saturday Fund (with which the Home is in connection) moved a resolution to make the forthcoming "Banner" service at the Pavilion Theatre a gathering worthy of the object which the meeting was called to support. Mr. Frewer announced that the Home was being enlarged so as to accommodate from twenty-four to thirty-six more beds. This will enable the Home to receive from 700 to 800 working men every year. Mr. George Noakes seconded the resolution, which was unanimously carried.

---

The special fund in aid of Guy's Hospital was assisted on Thursday by the proceeds of a performance at the Novelty Theatre, of Mr. Herman's adaptation of *Adrienne Lecouvreur*. To-night (Saturday) there will be a repetition of the performance. The cast includes the Hon. Lady Cadogan, Mrs. Hy. Morris, Mrs. Sutton Townsend, Mrs. Catesby Holland, Miss Selous, and Messrs. H. Cadogan, F. Evans, Harrison, C. Lloyd Mitford, and Ainsworth. The Prince and Princess of Wales, the Princess Frederica of Hanover, the Duchess of Teck, the Lord Mayor and Lady Mayoress, and many of the leading members of the aristocracy, accorded their patronage to the performance.

The Glasgow Medical Charities Committee appointed at the public meeting of practitioners, held in the Faculty Hall on the 30th ult., met on Friday afternoon, 22nd inst., and having appointed Professor James Morton, M.D., Chairman, Dr. Edward McMillan, Vice-Chairman, and the other office bearers and members of the executive, proceeded to consider the initial steps of dealing with the whole subject of the abuse of medical charities. Professor Gairdner strongly advised a conference with the Charity Organisation Society, as being in possession of the best possible information regarding the abuse of charitable institutions, and the most successful means of dealing with it. He was of opinion that while the outdoor cases should be seen for the first time, the services of that Society might be obtained for purposes of investigation. Dr. Erskine spoke of the importance of introducing the lay element into the committee, and it was proposed to invite the managers of the various medical charities to confer with the committee, and become members of it. Other important points were afterwards considered. The Secretary (Dr. Robb, Carlton Place, Glasgow), who was desired to obtain information from other towns in England and Scotland, where reform of charitable medical institutions has been effected or is under consideration, will be glad to receive information and suggestions.

---

A movement has just been set afloat in South London for the purpose of making a "local appeal" in aid of Guy's. As the hospital specially ministers to the needs of the vast population on the south side of the Thames, it is pleasant to see that such an endeavour is being made ; but the population is unfortunately mostly a very poor one, and we hardly dare hope that the admirable movement initiated by Dr. Serjeant—an old Guy's man—will be productive of any very considerable sum. The appeal made by Guy's has placed a very handsome sum in Mr. Lushington's hands, which certainly ought to go a long way towards placing this time-honoured institution on a sound financial footing. One hundred thousand pounds has been asked for, and unless some millionaire steps in at the eleventh hour to fill the gap, the treasurer of Guy's will not, we fear, be able to trouble Mr. Morgan for his princely donation.

---

The Foundling Hospital at Liverpool is a young institution which bids fair to do a very excellent work. The first step of a poor girl on a wrong course too often means her ultimate social ruin, unless some kind friend or institution chances to rescue her, and make her once more a respectable member of society. Conscious of her shame, and fearful of exposure, the expectant mother, weak in body and frenzied in mind, determines perhaps to destroy the evidence of her guilt. Infanticide in large centres of population is almost always due to this unhappy state of things. The object of the Liverpool Foundling Hospital is twofold :—(1) To afford poor girls who have fallen *only once* an opportunity for making a fresh start in life ; and (2) to provide a home for the children thus born. The ten months that the hospital has been established have been quite sufficient to prove the need there was for such an institution.

THE following sums have been recently received by the various Medical Charities, the name of the donor or the source from which they were obtained being given in each instance after the name of the Institution :—

**Donations.**

Bristol General Hospital. The Bedminster Coal Company £17 10*s.*
British Home for Incurables, Putney. Vestry of St. Leonards, Eastcheap, £10 10*s.*
City of London Dispensary. The Vestry of the Parish of St. Martin Ongar, £21.
Guy's Hospital. The Vestry of St. Martin Ongar, £21.
Home for Incurable Children, Maida Vale. Mrs. E. Armitage, £30.
Hospital for Epilepsy and Paralysis, etc., Regent's Park. Miss Fothergill, £15. Clothworkers' Company, £25.
London Hospital. The Vestry of St. Martin Ongar, £21.
National Eye and Ear Infirmary, Dublin. Messrs. A. Guinness, Sons and Co., £15.
North-Eastern Hospital for Children, Hackney Road. Vestry of St. Leonards, Eastcheap, £10 10*s.* Vestry of All Hallows, Lombard Street, £21.
North London Hospital. Mr. Charles Twamley, £10.
Queen Charlotte's Lying-in Hospital, Marylebone Road. Mr. James B. Deacon, £52 10*s.*
Sunderland Infirmary. The North-Eastern Marine Engineering Co., £11 12*s.* 11*d.* The Silksworth Colliery Company, £18 19*s.* 11*d.*
St. Barnabas Convalescent Home, Ramsgate. The Vestry of St. Martin Ongar, £10 10*s.*
West London Hospital, Hammersmith. Mrs. Gomonde, £10.

**Legacies.**

Cancer Hospital, Brompton. Mr. Benjamin Lancaster, £1,000.
Children's Hospital, Birmingham. Mr. W. Webb, £100. Mr. W. Middlemore, £90. Mr. C. Hollins, £75.
Consumption Hospital, Brompton. Mr. Benjamin Lancaster, £500.
Hospital for Incurables, Putney. Mr. Benjamin Lancaster, £500.
King's College Hospital. Mr. Benjamin Lancaster, £500.
Middlesex Hospital. Mr. Benjamin Lancaster, £500.
Nurses' Home, Norfolk Street, Strand. Mr. Benjamin Lancaster, £500.
St. George's Hospital, Hyde Park Corner. Mr. Benjamin Lancaster, £1,000.

**Entertainments.**

Children's Hospital, Birmingham. Part proceeds of Charity football match, £10. Theatre Royal Jubilee Hospital Benefit Fund, £27.
Croydon General Hospital. Gross proceeds of recent performance at Croydon Circus, per Mr. W. Rowland, proprietor, £15.

---

THE Committee of the Great Northern Central Hospital have received a donation of one thousand guineas (£1,050) to the building fund from Mr. R. A. Newbon, of Islington, to endow a bed in the new hospital in the Holloway Road in memory of his late sister. Of the £45,000 required to complete the new buildings only £10,000 has as yet been subscribed, and it is hoped therefore that the good example of Mr. Newbon will be followed by others, and the necessary funds will soon be provided.

---

THE present financial condition of the Pembrokeshire and Haverfordwest Infirmary is hardly one upon which the institution can be congratulated. As it is, there is a small balance due to the treasurer, and but for a timely windfall of £200, in the shape of a legacy from an old inhabitant of Haverfordwest, the result would have been disastrous. It would be interesting to learn how the dieting of the patients has risen from 6*s.* 1½*d.* per head per week under the old system, to 10*s.* 7*d.* under the new.

MISS FITZPATRICK, Matron of the Yeovil Hospital, has been unanimously elected Matron to the Royal Isle of Wight Infirmary, Ryde, for which latter post there were eighty-seven applicants. We congratulate Miss Fitzpatrick, and believe that the Ryde Hospital authorities will have every reason to be satisfied with the choice they have made.

---

WE are glad to be able to announce that we hope shortly to be in a position to afford our readers a full insight into the invaluable work which Lady Dufferin is doing for the women of India. Lady Dufferin writes to us from the Viceroy's Camp under date the 30th ult., that although she cannot at the present moment undertake to prepare articles herself, she has taken steps to procure and forward every information concerning this most excellent, useful, and praiseworthy movement.

---

CAPTAIN RAMSAY, to whose active benevolence and indefatigable zeal in the promotion of all charitable works the present cottage hospital at Norwood, amongst others, owes its existence, has not forgotten that useful little institution in his will. We are pleased to learn that he has bequeathed to it the sum of £100, although his generosity during his lifetime might be thought to have rendered any legacy unnecessary.

---

WE are informed that there are two ladies in responsible charge of the National Hospital for the Paralysed and Epileptic, Queen Square, the senior being called "Lady Superintendent," and her assistant "Superintendent of Nursing." Hence Miss Mollett was wrongly described as "Lady Superintendent," she having been until recently Superintendent of Nursing at the National Hospital, with which institution she has ceased to be connected. Our readers will have observed from Miss Mollett's contributions to this Journal that she is one of the ablest, most intelligent, and most efficient workers in the nursing field, and we know that any institution will be fortunate which secures her services in the immediate future.

---

THE Managers of the Potter's Bar Cottage Hospital, which is designed for the benefit of persons suffering from accidents or disease of a temporary character, have issued their third annual report. During the past year fifty-three patients have been treated in the hospital, of whom forty-three have been discharged cured or improved, whilst four cases terminated fatally. The use of the buildings is given rent free by Mr. Henry Parker, and the authorities have thus far remitted the payment of taxes, so that this hospital is exceptionally fortunate. The expenditure in 1886 amounted to £270, and the receipts (including payments by patients £33) to £321, and of the surplus £44 has been invested in 2½ per cent. stock. The outlay during the current year will probably exceed that of the past, as the hospital is about to be closed for certain necessary repairs and structural alterations. It is therefore hoped that the liberality hitherto shown by friends of the charity will be continued, and even increased.

